# Inter-examiner and intra-examiner reliability of optical coherence tomography angiography in vascular density measurement of retinal and choriocapillaris plexuses in healthy children aged 6–15 years

**DOI:** 10.3389/fmed.2023.1161942

**Published:** 2023-06-01

**Authors:** Kai Diao, Xiaomin Huang, Mingyu Yao, Jiawei Li, Feifei Fan, Hongxian Pan, Jinjin Yu, Yizhou Yang, Weiwei Lu, Hengli Lian, Qinmei Wang, Jinhai Huang, Ruru Chen

**Affiliations:** ^1^School of Ophthalmology and Optometry and Eye Hospital, Wenzhou Medical University, Wenzhou, China; ^2^Eye Institute and Department of Ophthalmology, Eye & ENT Hospital, Fudan University, Shanghai, China; ^3^NHC Key Laboratory of Myopia, Fudan University, Shanghai, China; ^4^Key Laboratory of Myopia, Chinese Academy of Medical Sciences, Beijing, China; ^5^Shanghai Research Center of Ophthalmology and Optometry, Shanghai, China

**Keywords:** capillary plexus, children, foveal avascular zone, macula, optical coherence tomography angiography, vascular density

## Abstract

**Objective:**

This study aimed to test the inter-examiner and intra-examiner reliability of macular vascular density (VD) measurement of retinal and choriocapillaris plexuses in healthy children using optical coherence tomography angiography (OCTA).

**Materials and methods:**

Ninety-two school children were prospectively recruited. Macular OCTA images (6 × 6 mm^2^) were obtained thrice by two examiners using the RTVue-XR Avanti OCT system. The coefficient of variation (COV), intraclass correlation coefficient (ICC), and Bland–Altman plots were used to evaluate the repeatability and reproducibility.

**Results:**

Ninety participants aged 6–15 years were enrolled; two participants were excluded because of low-quality images. In the retina, the reproducibility and repeatability of VD became poorer from superficial to deep retinal capillary plexus (superficial: COV = 4.61–11.11%; intermediate: COV = 7.73–14.15%; deep: COV = 14.60–32.28%). For both reproducibility and repeatability, the ICC ranged from moderate to high (superficial plexus: ICC = 0.570–0.976; intermediate plexus: ICC = 0.720–0.968; deep plexus: ICC = 0.628–0.954). In the choroid, the inter-examiner reproducibility and intra-examiner repeatability of the VD measurement of choriocapillaris were excellent in the macula, fovea, parafovea, and perifovea (COV = 1.00–6.10%; ICC = 0.856–0.950). The parameters of the foveal avascular zone (FAZ) also showed significant reproducibility and repeatability (COV = 0.01–0.21%; ICC = 0.743–0.994).

**Conclusion:**

The VD measurements of the choriocapillaris and FAZ parameters using OCTA showed excellent inter-examiner and intra-examiner reliability in school children. The reproducibility and repeatability of the VD of three retinal capillary plexuses depended on the depth of the retinal capillary plexus.

## Introduction

Optical coherence tomography angiography (OCTA) is a noninvasive and quick imaging technique to enable the visualization of retinal microvasculature without fluorescein dye injection. It has been widely applied in the diagnostic and pathogenetic studies of several ocular diseases including ischemic maculopathy (1), age-related macular degeneration (2), diabetic retinopathy (3), myopia (4), Marfan syndrome (5), and so on.

During follow-up, OCTA demonstrates high short-and long-term repeatability and reproducibility in measuring the foveal avascular zone (FAZ) and vascular density (VD) of adults with pathologic eyes (6–10). Some recent studies used OCTA to examine ocular diseases involving vascularization or vessel changes in children (11). Gołębiewska et al. (12) and Cheung et al. (13) found on the OCTA examination that the retinal superficial VD was lower in myopic than in emmetropic children, and the area of FAZ was larger in myopic than in emmetropic children. Many studies investigated the correlation between the mechanism of myopia progression and the parameters of OCTA (12, 13).

Evaluating the repeatability and reproducibility of OCTA in a pediatric sample was essential considering that children might not cooperate as effectively as adults during an examination. In this study, the macular VD in the superficial, intermediate, and deep retinal capillary plexuses (SCP, ICP, and DCP, respectively) and the choriocapillaris (CC) were measured using OCTA. A customized segmentation method was used to evaluate the inter-and intra-examiner reliability of OCTA in healthy children aged 6–15 years.

## Materials and methods

### Study participants

This study was designed to evaluate the inter-examiner and intra-examiner reliability of OCTA in healthy children. Written informed consent was obtained from the legal guardians of the children prior to enrollment. This study was conducted following the tenets of the Declaration of Helsinki and approved by the ethics committee of the Eye Hospital of Wenzhou Medical University.

Ninety-two patients aged 6–15 years were prospectively recruited from the Eye Hospital of Wenzhou Medical University for this study. The inclusion criteria included the following: (1) aged 6–15 years, (2) best-corrected visual acuity of 20/25 or better, (3) intraocular pressure (IOP) <21 mm Hg, and (4) avoiding caffeine for 12 h prior to the study. The exclusion criteria involved the following: (1) having systemic or ocular diseases that might affect the ocular circulation, such as glaucoma or diabetes mellitus and (2) exhibiting any sign of pathological myopia at a funduscopic examination. Each patient underwent a standard ophthalmological examination, including cycloplegic autorefraction (ARK-1; Nidek, Japan), air puff IOP (NT-510; Nidek), and axial length (AL) measurement (IOLMaster 500; Carl Zeiss, Germany). All patients enrolled in this study were without retinal pathology or any history of systemic disease.

### Image acquisition and OCTA analysis

The macular OCTA images were captured using the RTVue-XR Avanti OCT system (Optovue, CA, United States) and RTVue software (Optovue) with the Angio Retina HD (6 × 6 mm^2^) mode. Given the recent improvement in retinal segmentation and the development of algorithms removing projection artifacts originating from the superficial layers (14), OCTA allows the identification of three distinct retinal vasculature capillary plexuses: SCP, ICP, and DCP, which replicates the retinal structure revealed by histological studies in humans. The three-layer segmentation method is believed to better reflect the real status of the microvasculature. Some attempts have been made to explore the association of retinal VD with ocular disease progression using the new segmentation method (e.g., diabetic retinopathy) (15). The concrete custom boundaries of the three retinal layers were defined in previous studies by Lavia et al. (14, 16) as follows: (1) SCP was set between the inner limiting membrane and 9 μm above the junction between the inner plexiform layer and the inner nuclear layer (IPL–INL), (2) ICP was set between 9 μm above the IPL–INL junction and 6 μm below the inner nuclear layer and the outer plexiform layer (INL–OPL) junction, and (3) DCP extended from 6 μm below the INL–OPL junction to 9 μm below OPL and outer nuclear layer junction. The CC slab was autosegmented from 9 μm above the Bruch’s membrane (BRM) to 31 μm beneath the BRM. In most previous studies, the retinal vasculature was generally segmented into two layers of blood vessels using commercial OCTA instruments: SCP and deep vascular complex (DVC) (17).

The VD was defined as the ratio of the area of the large vessel and capillary vessel divided by the total area measured in particular sections. We measured the VDs in the whole macular, foveal, parafoveal, and perifoveal regions. En face OCTA images were automatically segmented into fovea, parafovea, and perifovea using the software. The “fovea” refers to the central 1-mm zone. In accordance with the Early Treatment Diabetic Retinopathy Study (ETDRS), the parafovea and perifovea regions were divided into four quadrants (i.e., superior, inferior, temporal, and nasal) or two equal hemispheres (superior and inferior). The VD in each ETDRS region was also measured.

In the image scan, the area of FAZ was automatically in mm^2^, outlined, and calculated with this software using the FAZ measurement function. The perimeter and acircularity index of FAZ were also calculated. Foveal VD 300 (FD-300) was defined as VD in a 300-mm wide zone around FAZ combining the SCP and DVC. The area density and length density of FD-300 were automatically calculated using the software and analyzed in this study.

**Figure 1:**
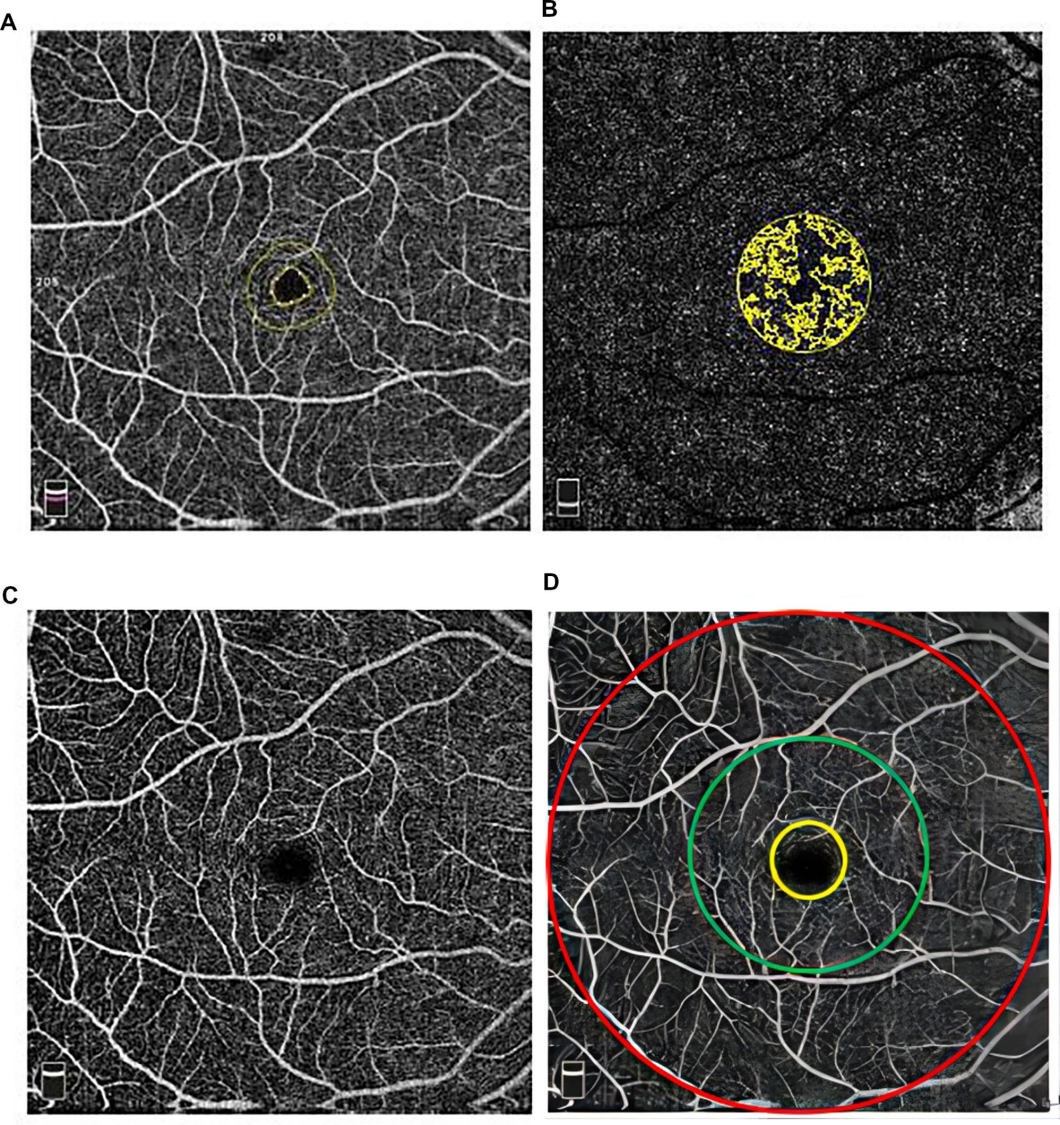
Images of **(A)** foveal avascular zone, **(B)** deep capillary layer (yellow color represents foveal vd300 zone), **(C)** macular region, and **(D)** whole retina: the area inside the yellow circle represents the foveal zone, between the yellow and green circles represents parafovea and between the green and red circles represents perifovea of retina.

The retinal and choroidal OCTA images of the right eye were collected three times by two examiners with an interval of 5 min to assess the intra-examiner repeatability and inter-examiner reproducibility: first time by examiner 1, second time by examiner 2, and third time by examiner 1 again. All OCTA images were checked by one expert for the quality and correctness of automated layer segmentation. Images with motion artifacts, segmentation errors, or quality images on OCTA (signal strength index lower than 60 or quality index lower than 7) were excluded (Figure 1).

### Statistical analysis

Statistical analysis was performed using SPSS Statistics 25.0 software (IBM corporation, United States). A sample Kolmogorov–Smirnov test was used to verify the normality of these measured parameters. Data with a normal distribution were presented as the mean ± standard deviation (SD). The inter-examiner reproducibility was defined as the agreement in measurements between two images taken at the same eye using the same device but processed by two different examiners (examiner 1 vs. examiner 2). The intra-examiner repeatability was defined as the repeatability in quantitative measurements between two images captured of the same eye using the same device and processed by the same examiner.

The coefficient of variation (COV) and intraclass correlation coefficient (ICC) were used to evaluate the repeatability and reproducibility. The COV value represented the dispersion of the measures. COV was referred to as the within-patient SD (the square root of the residual mean square from the repeated-measures analysis of variance) divided by the average measurement. A lower COV value indicated higher repeatability, and devices with a COV less than 10% showed high repeatability, with a COV value less than 5% indicating a higher degree of repeatability (16). The ICC represented the concordance between the results of the two examinations. An ICC value higher than 0.9 or lower than 0.5 indicated that the reproducibility of two repeated measurements was excellent or poor (18). Agreement among the two measurements was analyzed using the Bland–Altman plots, which displayed the association of the difference between the two examinations and their mean. The 95% limits of agreement (LoAs) were calculated by the difference ± 1.96 times the SD of the differences.

## Results

Initially, 92 healthy children were enrolled in this prospective study. Two children were excluded due to low-quality images. Therefore, the study finally included 90 Chinese children (45 boys and 45 girls) with 90 eyes whose mean age was 9.40 ± 1.87 years (range: 6–15 years) (Table 1). The mean AL was 24.72 ± 1.26 mm. The mean spherical equivalent refraction was −2.72 ± 1.94 D (range: −6.63–+1.50 D).

**Table 1:** The age distribution of subjects.

Age (years)	6–7	8–9	10–11	12–13	14–15
Number (N)	10	46	21	11	2
Percentage (%)	11.11	51.11	23.33	12.22	2.22

### Inter-examiner variability

**Figure 2:**
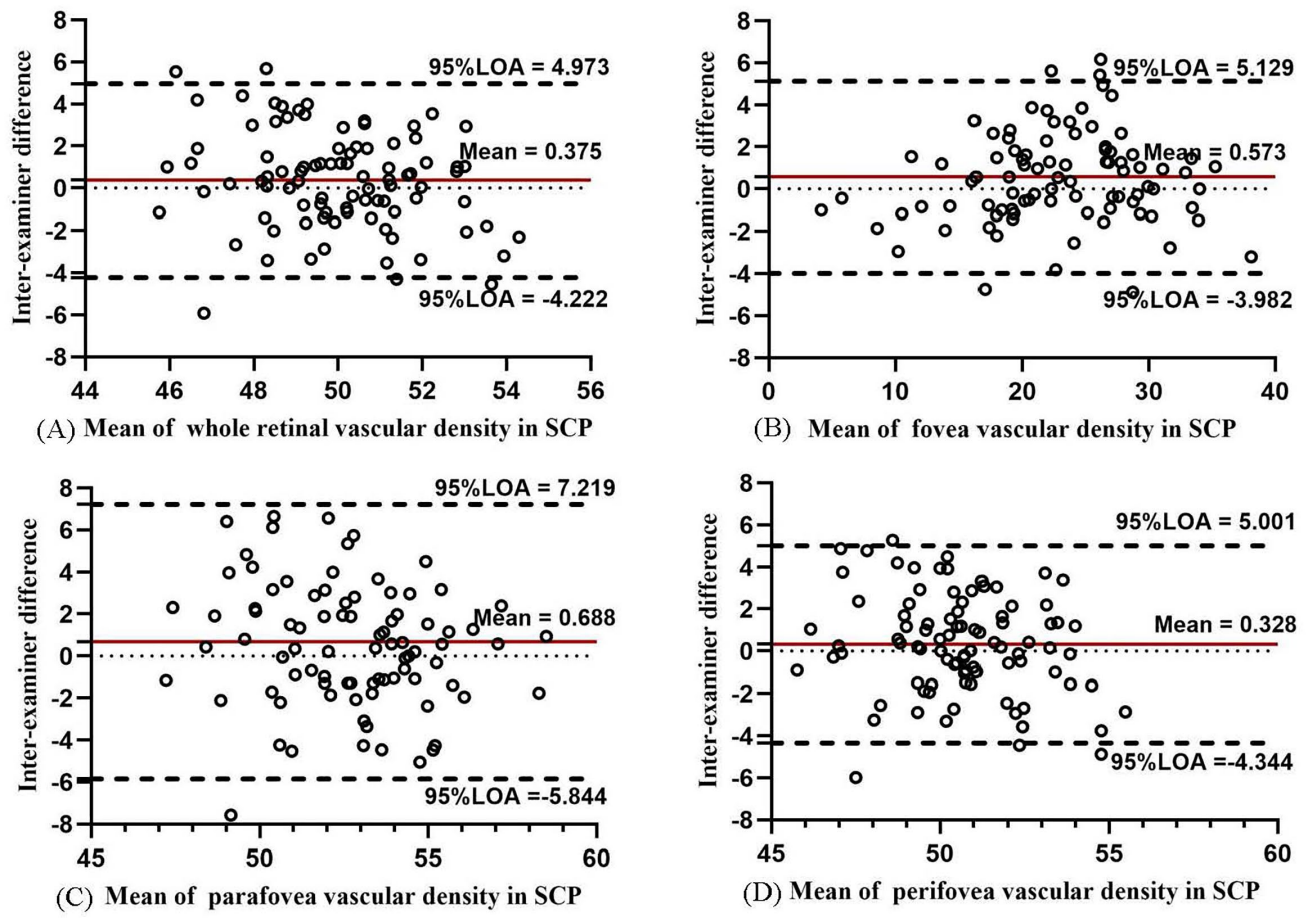
Bland–Altman plots showing agreement in results between two examiners measuring vascular density for SCP in the whole macula **(A)**, fovea **(B)**, parafovea **(C)**, and perifovea **(D)**.

In SCP, the VD of SCP in the whole macula, parafovea, and perifovea was about 50% and that of the fovea was 22.668% (Table 2). The inter-examiner COV in the macula, fovea, parafovea, and perifovea was 4.61, 9.42, 9.36, and 4.68%, with the ICC values of 0.741, 0.976, 0.646, and 0.762, respectively. The Bland–Altman plots showed good agreement for the VD in SCP between the two examiners (all 95% LoAs within −5.844 to 7.219% for the SCP of the whole macula, parafovea, perifovea, and fovea; Figure 2). The inter-examiner COV and ICC values in the temporal, superior, nasal, and inferior regions of parafoveal and perifoveal sectors are detailed in Supplementary Table S2. The inter-examiner reproducibility of the SCP measurements in each ETDRS sector was moderate. The intra-examiner repeatability also showed similar reliability to reproducibility.

**Table 2:** Inter-examiner variability of the OCTA in measuring vascular density of SCP, ICP, DCP, and CC in macular.

		Whole retina	Fovea	Parafovea	Perifovea
SCP	Mean ± SD (%)	49.934 ± 2.362	22.668 ± 6.728	52.536 ± 3.083	50.570 ± 2.473
	COV (%)	4.61	9.42	9.36	4.68
	ICC (95% CI)	0.741 (0.607, 0.829)	0.976 (0.964, 0.984)	0.646 (0.464, 0.766)	0.762 (0.638, 0.843)
ICP	Mean ± SD (%)	43.967 ± 3.830	38.550 ± 6.895	48.482 ± 3.304	44.448 ± 4.026
	COV (%)	12.59	7.73	9.43	14.15
	ICC (95% CI)	0.767 (0.647, 0.846)	0.968 (0.951, 0.979)	0.736 (0.599, 0.826)	0.760 (0.636, 0.842)
DCP	Mean ± SD (%)	55.015 ± 4.764	35.155 ± 7.636	54.676 ± 5.676	56.373 ± 5.353
	COV (%)	19.23	31.87	32.28	21.45
	ICC (95% CI)	0.701 (0.544, 0.803)	0.895 (0.840, 0.931)	0.628 (0.434, 0.755)	0.737 (0.599, 0.827)
CC	Mean ± SD (%)	70.312 ± 2.693	71.493 ± 4.103	68.240 ± 3.514	71.103 ± 2.711
	COV (%)	1.11	3.94	2.91	1.15
	ICC (95% CI)	0.944 (0.915, 0.963)	0.910 (0.863, 0.9413)	0.914 (0.869, 0.9433)	0.942 (0.912, 0.9623)

SCP, superficial capillary plexus; ICP, intermediate capillary plexus; DCP, deep capillary plexus; CC, choroidal capillary plexus.

**Figure 3:**
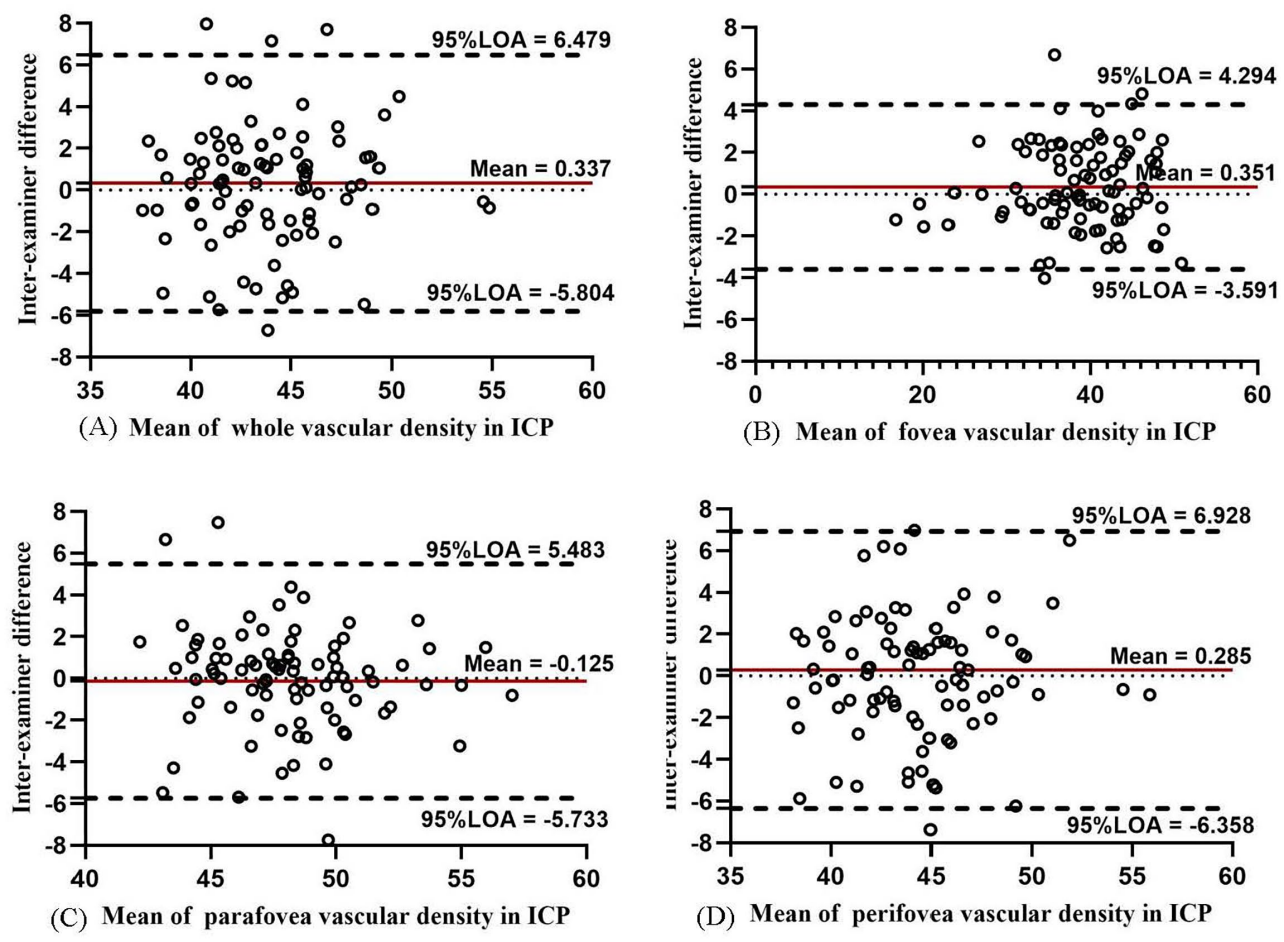
Bland–Altman plots showing agreement in results between two examiners measuring vascular density for ICP in the whole macula **(A)**, fovea **(B)**, parafovea **(C)**, and perifovea **(D)**.

**Figure 4:**
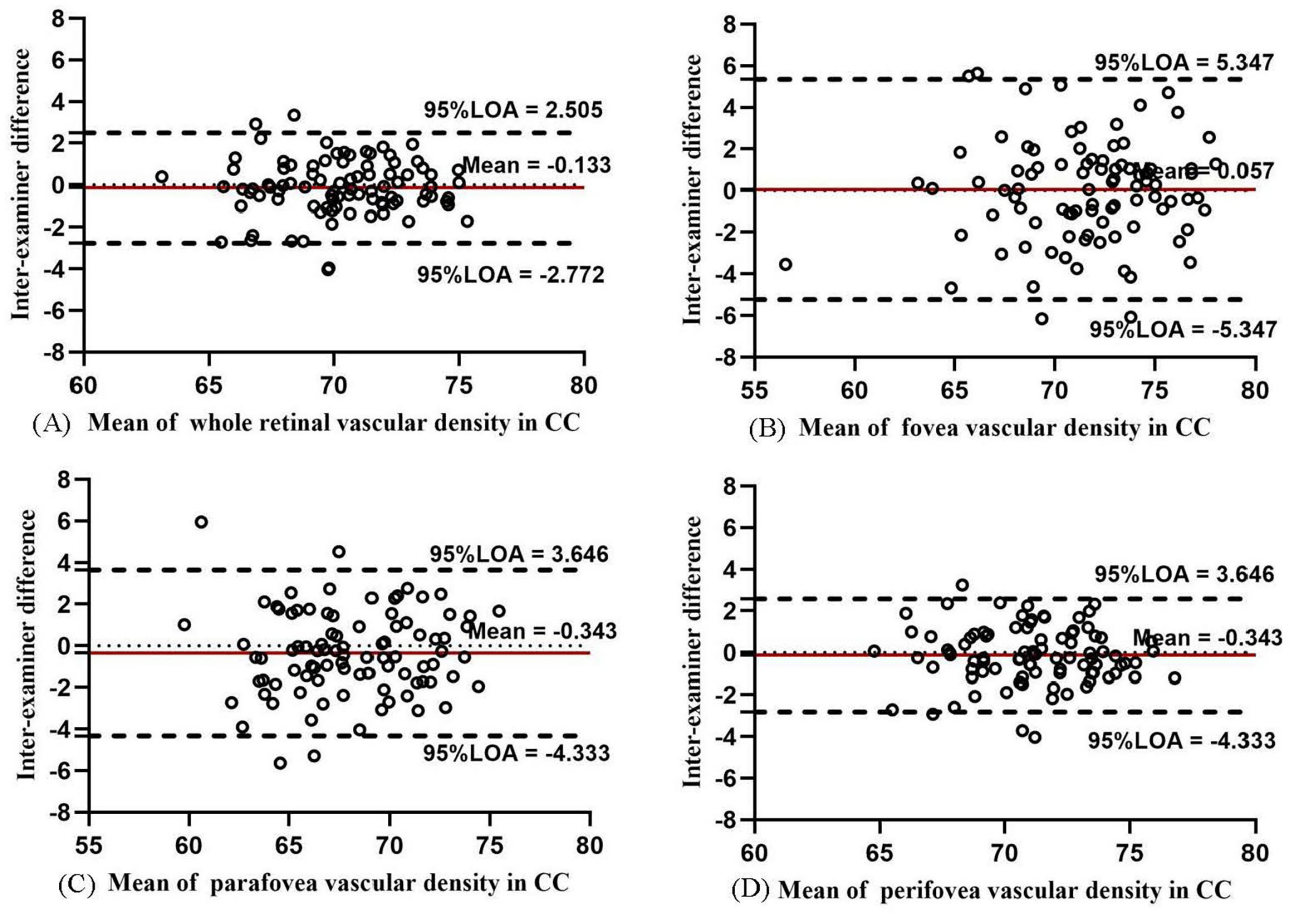
Bland–Altman plots showing agreement in results between two examiners measuring vascular density for DCP in the whole macula **(A)**, fovea **(B)**, parafovea **(C)**, and perifovea **(D)**.

The inter-examiner reproducibility of VD measurements in ICP and DCP was lower with respect to that of SCP. The inter-examiner COV value of ICP in the whole macula, fovea, parafovea, and perifovea was 12.59, 7.73, 9.43, and 14.15%, respectively. In DCP, the inter-examiner COV values increased to 19.23, 31.87%, 32.28, and 21.45%, respectively. The inter-examiner reproducibility was moderate in ICP and DCP (ICC = 0.767, 0.968, 0.736, and 0.760 in ICP and ICC = 0.701, 0.895, 0.628, and 0.737 in DCP). The Bland–Altman plots in ICP (Figure 3) showed good agreement between two examiners (all 95% LoAs within −6.358 to 6.928% in ICP of the whole macula, parafovea, perifovea, and fovea), similar to the agreement in SCP. The Bland–Altman plots in DCP showed poor agreement between two examiners (all 95% LoAs within −11.28 to 11.39% in DCP; Figure 4). The intra-examiner repeatability also showed similar reliability to reproducibility.

**Figure 5:**
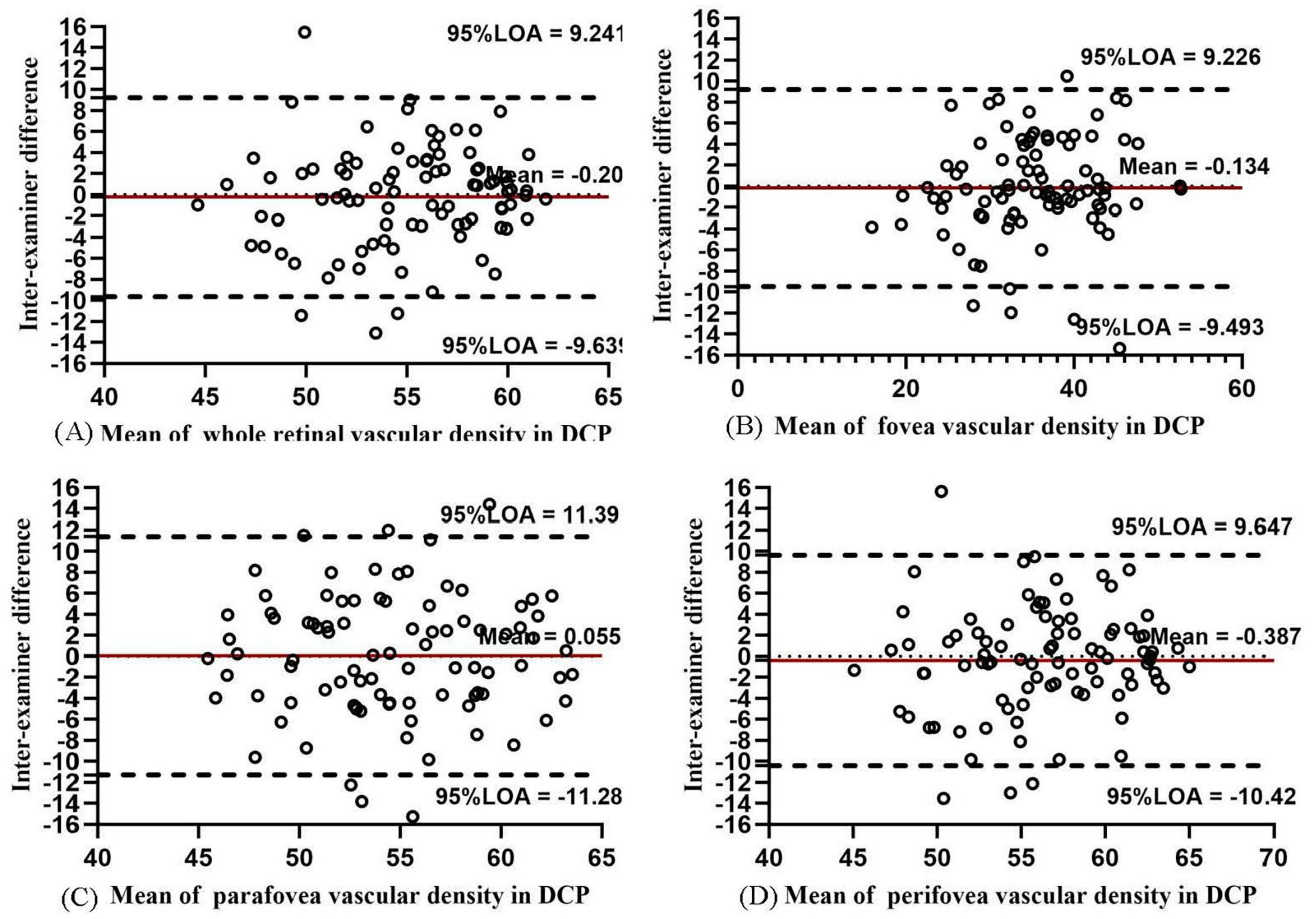
Bland–Altman plots showing the inter-examiner agreement in measuring vascular density for CC in the whole macula **(A)**, fovea **(B)**, parafovea **(C)**, and perifovea **(D)**.

In CC, the VD values in the macula, fovea, parafovea, and perifovea were the highest among the four layers (from 68.240 to 71.493%; Table 2). The inter-examiner COV value was 1.11, 3.94, 2.91, and 1.15% in the macula, fovea, parafovea, and perifovea, respectively. The ICC value of the aforementioned four areas was 0.944, 0.910, 0.914, and 0.942, respectively, ranking “excellent.” The Bland–Altman plots (Figure 5) showed high agreement between different examiners (all 95% LoAs within −5.347 to 5.404% in CC). The inter-examiner reproducibility and intra-examiner repeatability of the VD measurements in CC in each ETDRS sector also showed good agreement (Supplementary Tables S1, S2).

**Figure 6:**
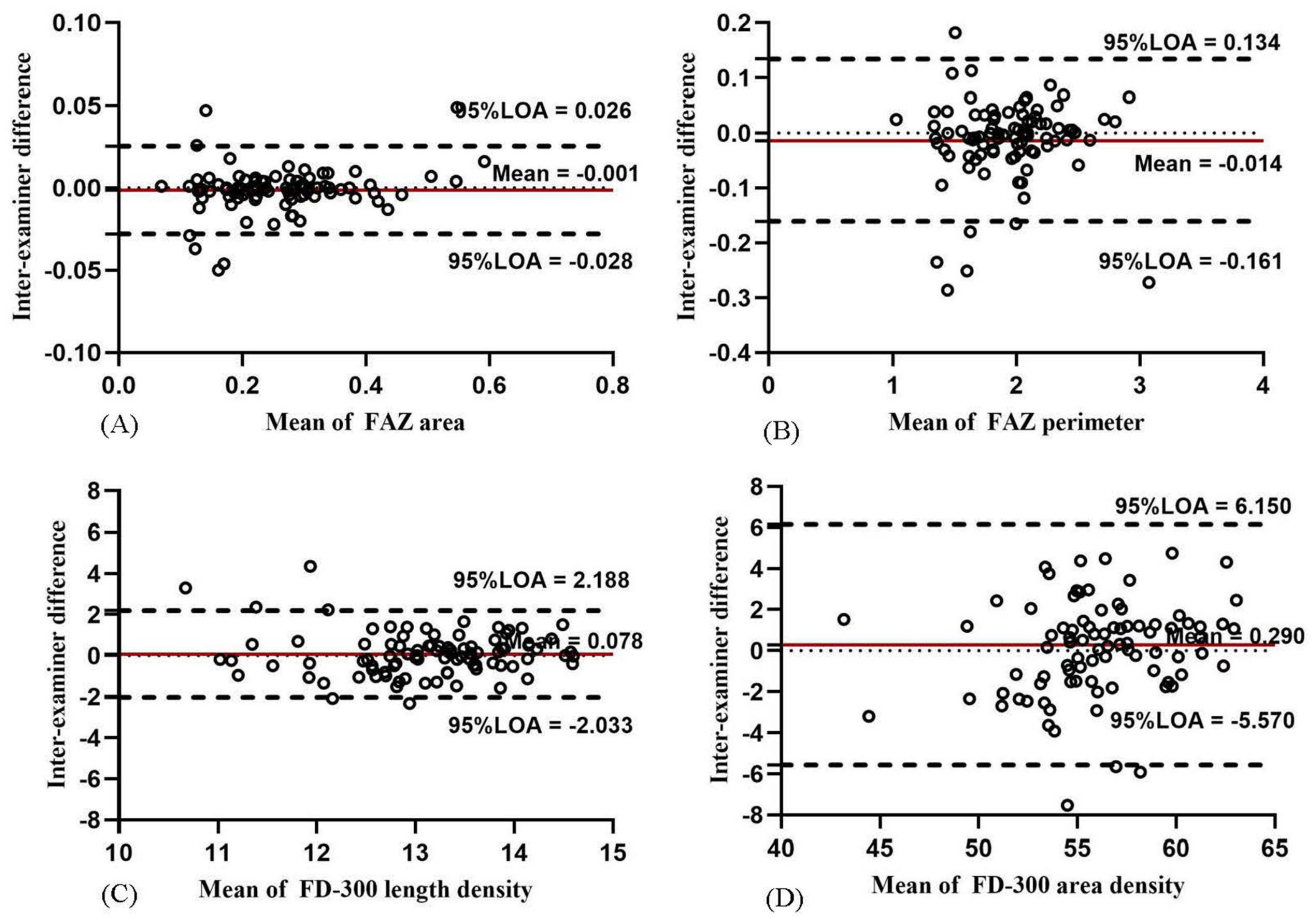
Bland–Altman plots showing the inter-examiner agreement for the parameters of FAZ and FD-300 in macula: agreement for FAZ area **(A)**, FAZ perimeter **(B)**, FD-300 length density **(C)**, and FD-300 area density **(D)**.

For FAZ-related parameters, the inter-examiner reproducibility of FAZ and FD-300 is presented in Table 3. The FAZ, perimeter, and acircularity index showed good reproducibility (all COV values <0.21% and ICC = 0.861–0.986). The Bland–Altman plots (Figures 6A,B) also showed good agreement.

**Table 3:** Inter-examiner variability of the parameters of FAZ and FD-300 in macular.

Section	Mean ± SD	COV (%)	ICC (95% CI)
FAZ area (mm^2^)	0.261 ± 0.104	<0.001	0.994 (0.991, 0.996)
FAZ perimeter (mm)	1.924 ± 0.383	0.208	0.986 (0.978, 0.991)
FAZ acircularity index	1.085 ± 0.027	<0.001	0.861 (0.789, 0.908)
FD-300 area density (%)	55.973 ± 4.085	6.744	0.874 (0.809, 0.917)
FD-300 length density (%)	13.113 ± 1.047	3.798	0.710 (0.559, 0.809)

FAZ, foveal avascular zone; FD-300, foveal VD 300.

For FD-300-related parameters, FD-300 area density and length density showed moderate reproducibility (COV = 3.798–6.744% and ICC = 0.710–0.874). The Bland–Altman plots (Figures 6C,D) also showed moderate agreement.

### Intra-examiner variability

**Figure 7:**
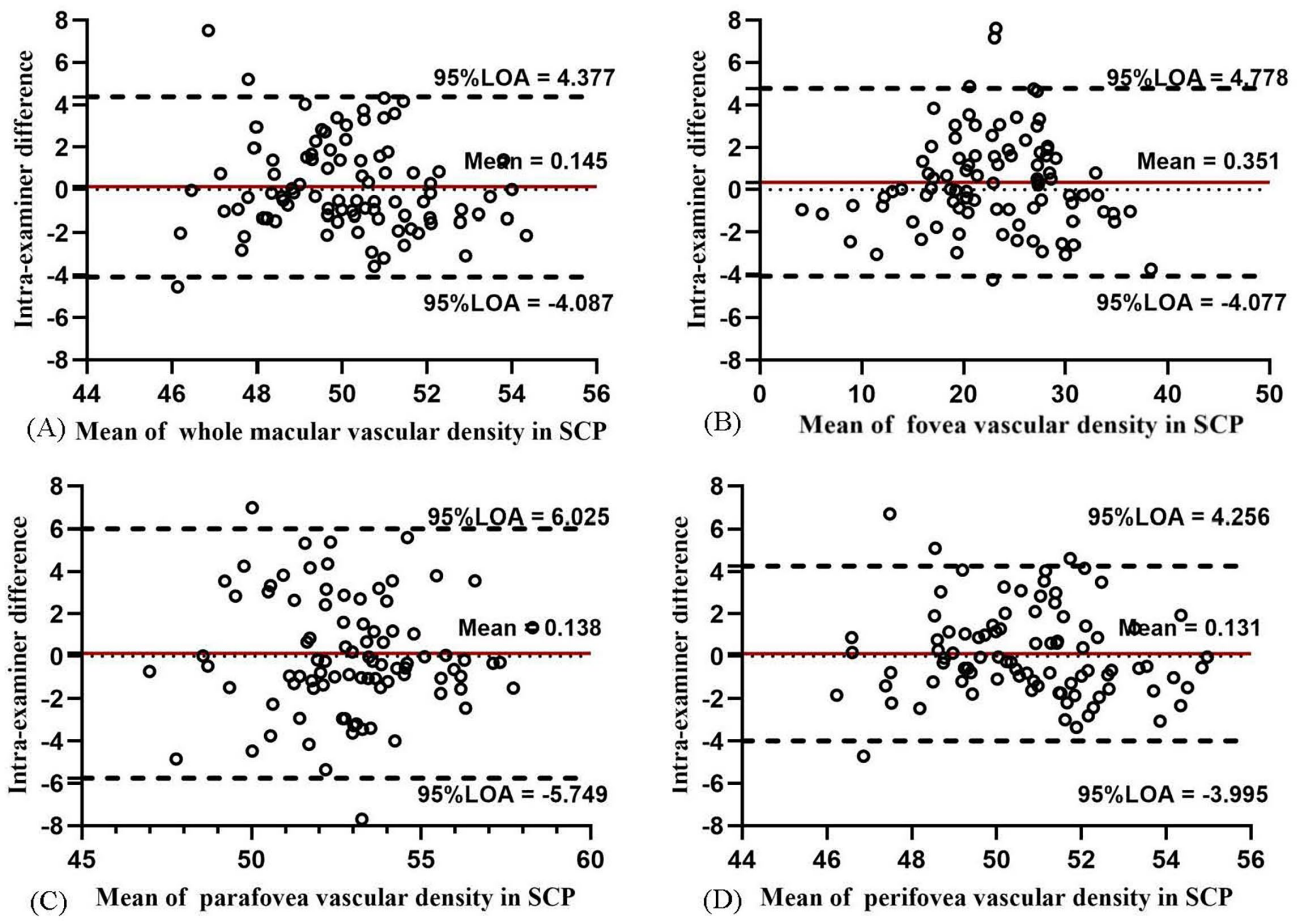
Bland–Altman plots showing the intra-examiner agreement in vascular density for SCP in the whole macula **(A)**, fovea **(B)**, parafovea **(C)**, and perifovea **(D)**.

In SCP, the intra-examiner variability between two examinations showed moderate repeatability in the whole macula, fovea, parafovea, and perifovea (COV = 4.65, 11.11, 8.53, and 4.37%; ICC = 0.655, 0.972, 0.570, and 0.723, respectively). The Bland–Altman plots found moderate-to-good intra-examiner agreement (all 95% LoAs within −5.749 to 6.025% in SCP in the whole macula, parafovea, perifovea, and fovea; Figure 7). The intra-examiner repeatability of the SCP measurements in each ETDRS sector was also moderate (Supplementary Table S3).

**Figure 8:**
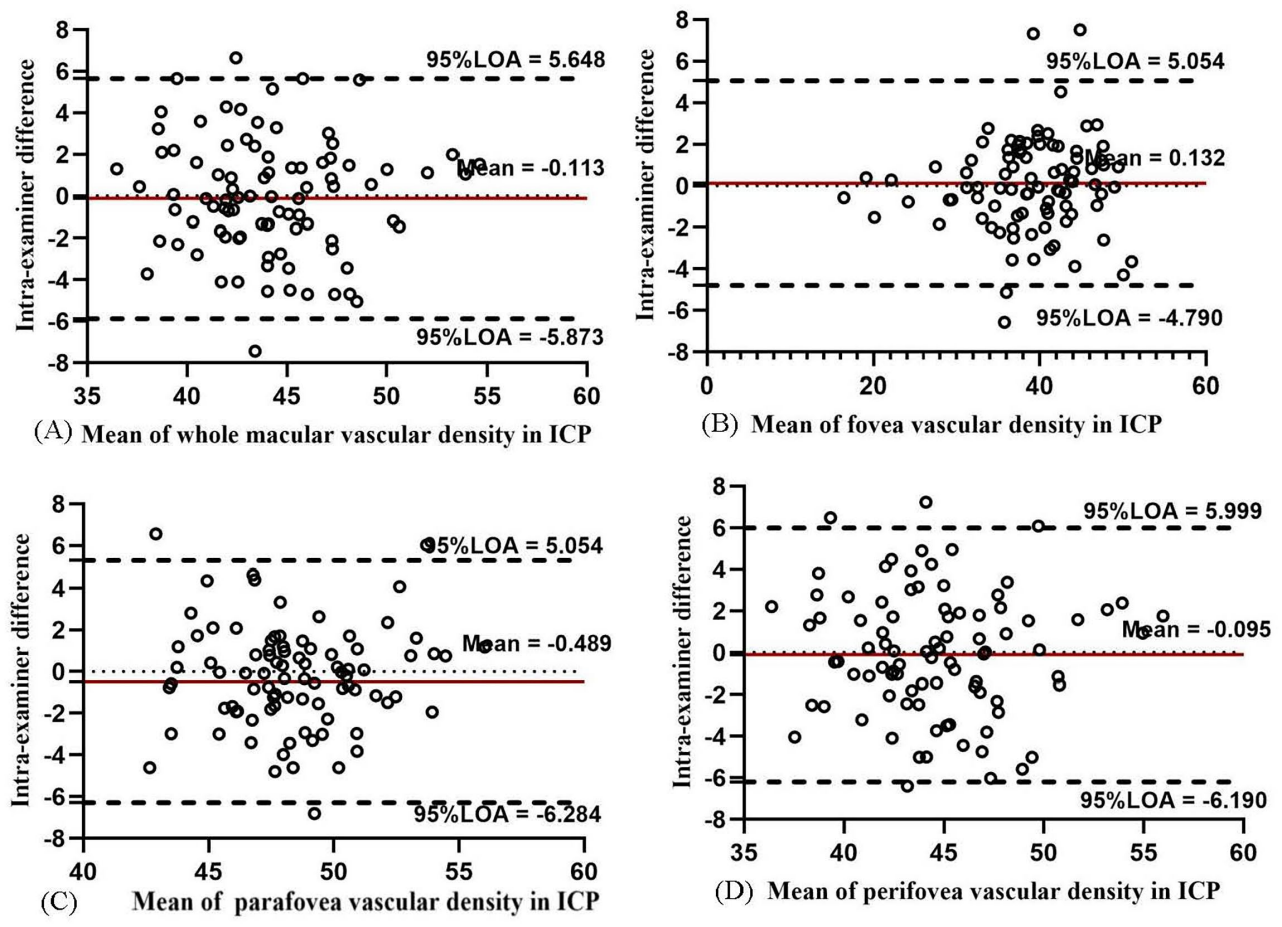
Bland–Altman plots showing the intra-examiner agreement in vascular density for ICP in the whole macula **(A)**, fovea **(B)**, parafovea **(C)**, and perifovea **(D)**.

**Figure 9:**
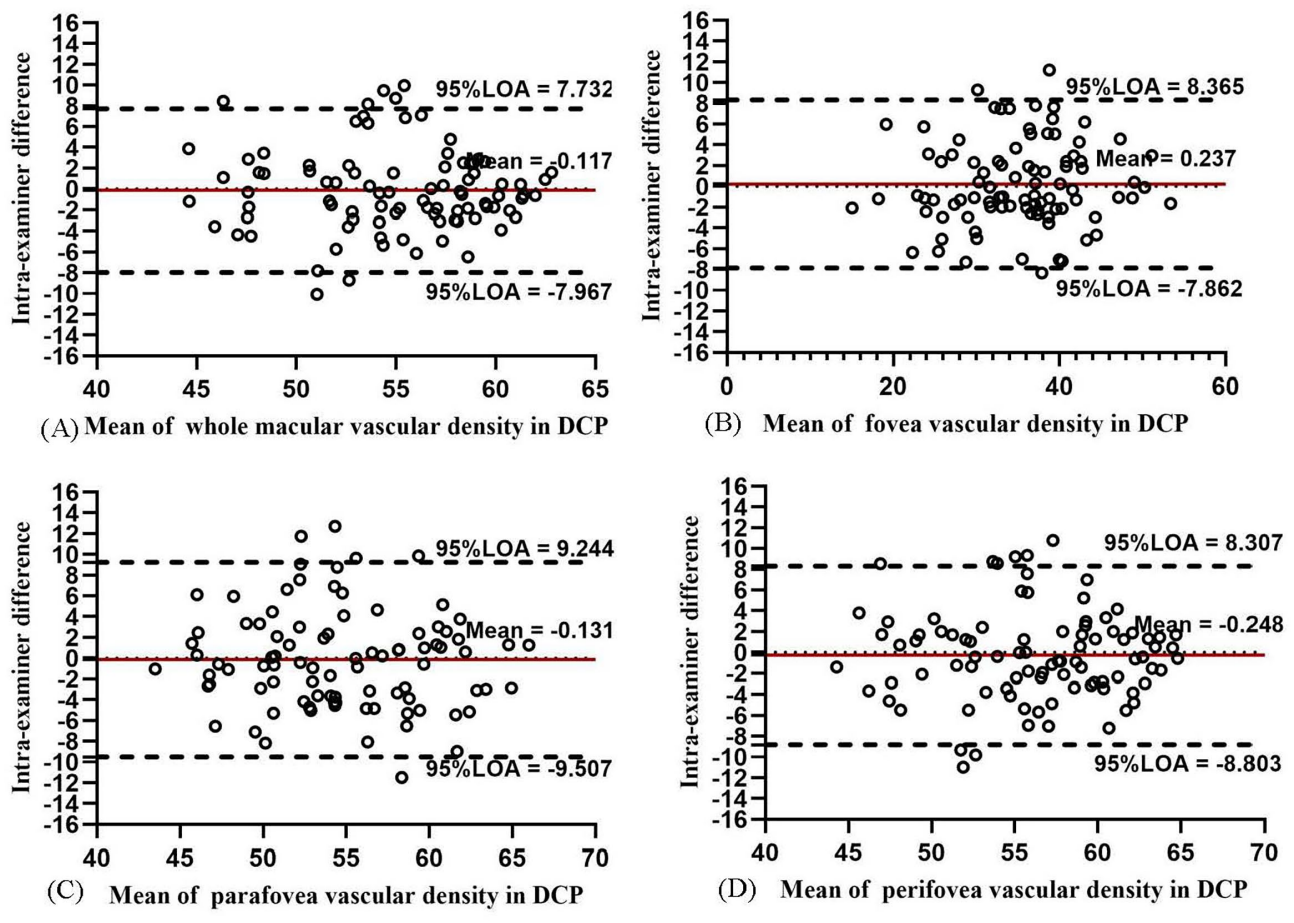
Bland–Altman plots showing the intra-examiner agreement in vascular density for DCP in the whole macula **(A)**, fovea **(B)**, parafovea **(C)**, and perifovea **(D)**.

The intra-examiner repeatability of VD measurements in ICP was relatively less repeatable than that in SCP, while the intra-examiner reproducibility of DCP was the worst among the four layers. In ICP, the intra-examiner COV value was 9.79, 8.14, 9.03, and 10.84% in the whole macula, parafovea, perifovea, and fovea, respectively. In DCP, the intra-examiner COV values increased to 14.60, 24.33, 20.91, and 16.96%, respectively. The intra-examiner ICC value was 0.842, 0.967, 0.720, and 0.842 in ICP and 0.807, 0.928, 0.788, and 0.821 in DCP, respectively. The Bland–Altman plots in ICP (Figure 8) and DCP (Figure 9) showed good agreement (all 95% LoAs within −6.284 to 5.99% in ICP in the whole macula, parafovea, perifovea, and fovea; all 95% LoAs within −9.507 to 9.244% in DCP in the same areas).

**Figure 10:**
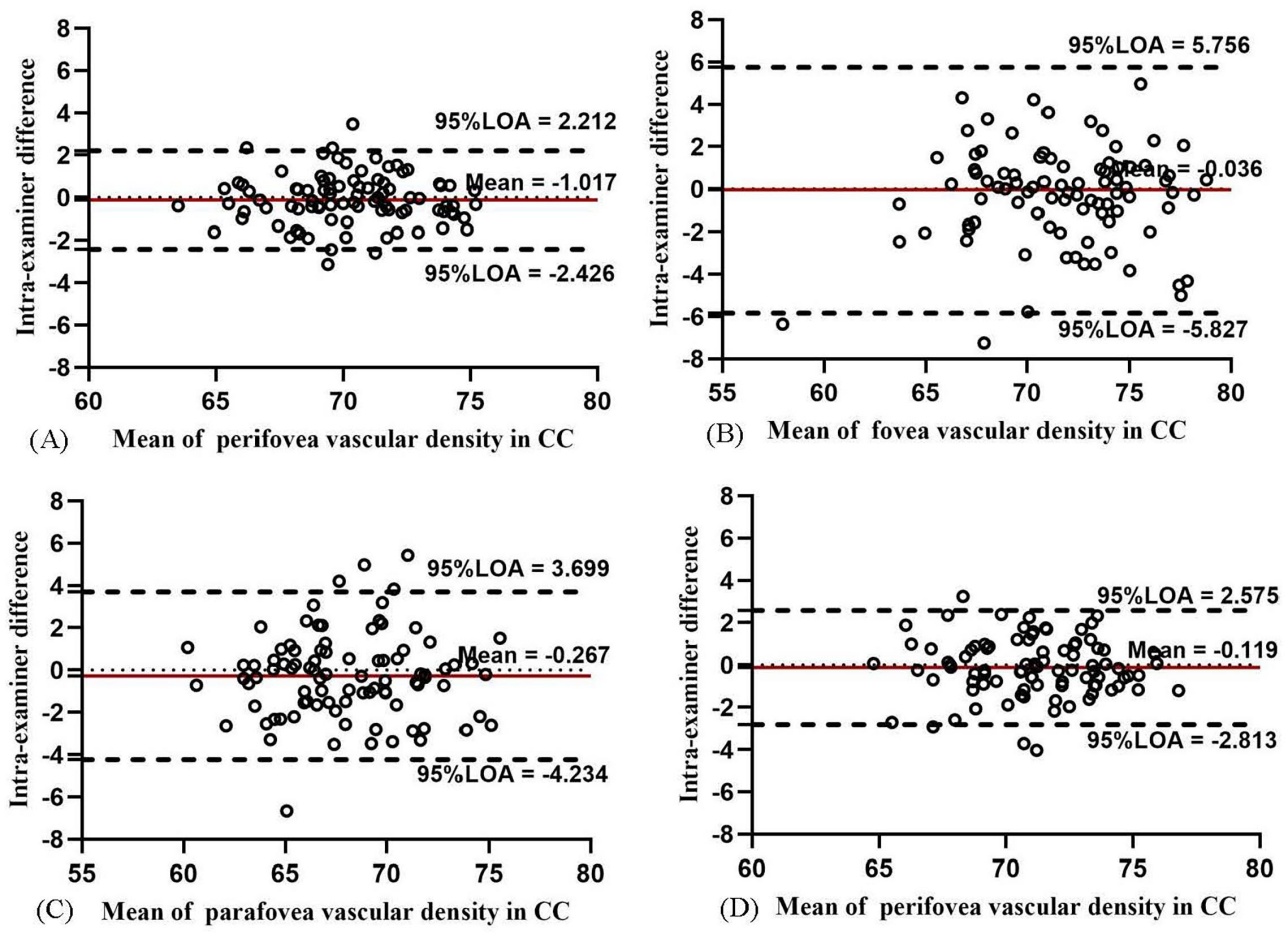
Bland–Altman plots showing the intra-examiner agreement in vascular density for CC in the whole macula **(A)**, fovea **(B)**, parafovea **(C)**, and perifovea **(D)**.

In CC, the intra-examiner repeatability of the VD measurements in the whole macula, fovea, parafovea, and perifovea was the highest among the four layers (Table 4). The intra-examiner COV value was 1.00, 6.10, 3.01, and 1.05% in the four areas, respectively. The ICC of the aforementioned four areas was about 0.9, indicating high repeatability. A high agreement was indicated in the Bland–Altman plot as most of the data points were at or within −5.827 to 5.756 (Figure 10). The intra-examiner VD measurement in CC in each ETDRS sector also showed high repeatability (Supplementary Table S3).

**Table 4:** Intra-examiner variability of the OCTA in measuring vascular density of SCP, ICP, DCP, and CC in macular.

		Whole retina	Fovea	Parafovea	Perifovea
SCP	Mean ± SD (%)	50.12 ± 2.34	22.95 ± 6.85	52.88 ± 2.73	50.73 ± 2.25
	COV (%)	4.65	11.11	8.53	4.37
	ICC (95% CI)	0.655 (0.476, 0.773)	0.972 (0.957, 0.981)	0.570 (0.345, 0.717)	0.723 (0.579, 0.818)
ICP	Mean ± SD (%)	44.135 ± 3.958	38.726 ± 6.975	48.419 ± 3.174	44.591 ± 4.181
	COV (%)	9.79	8.14	9.03	10.84
	ICC (95% CI)	0.842 (0.760, 0.896)	0.967 (0.950, 0.978)	0.720 (0.575, 0.815)	0.842 (0.760, 0.896)
DCP	Mean ± SD (%)	54.916 ± 4.953	35.088 ± 7.918	54.703 ± 5.685	56.179 ± 5.570
	COV (%)	14.60	24.33	20.91	16.96
	ICC (95% CI)	0.807 (0.707, 0.873)	0.928 (0.891, 0.953)	0.788 (0.678, 0.861)	0.821 (0.728, 0.882)
CC	Mean ± SD (%)	70.246 ± 2.704	71.521 ± 4.131	68.068 ± 3.557	71.043 ± 2.689
	COV (%)	1.00	6.10	3.01	1.05
	ICC (95% CI)	0.950 (0.924, 0.967)	0.856 (0.780, 0.905)	0.912 (0.866, 0.942)	0.947 (0.919, 0.965)

SCP, superficial capillary plexus; ICP, intermediate capillary plexus; DCP, deep capillary plexus; CC, choroidal capillary plexus.

**Figure 11:**
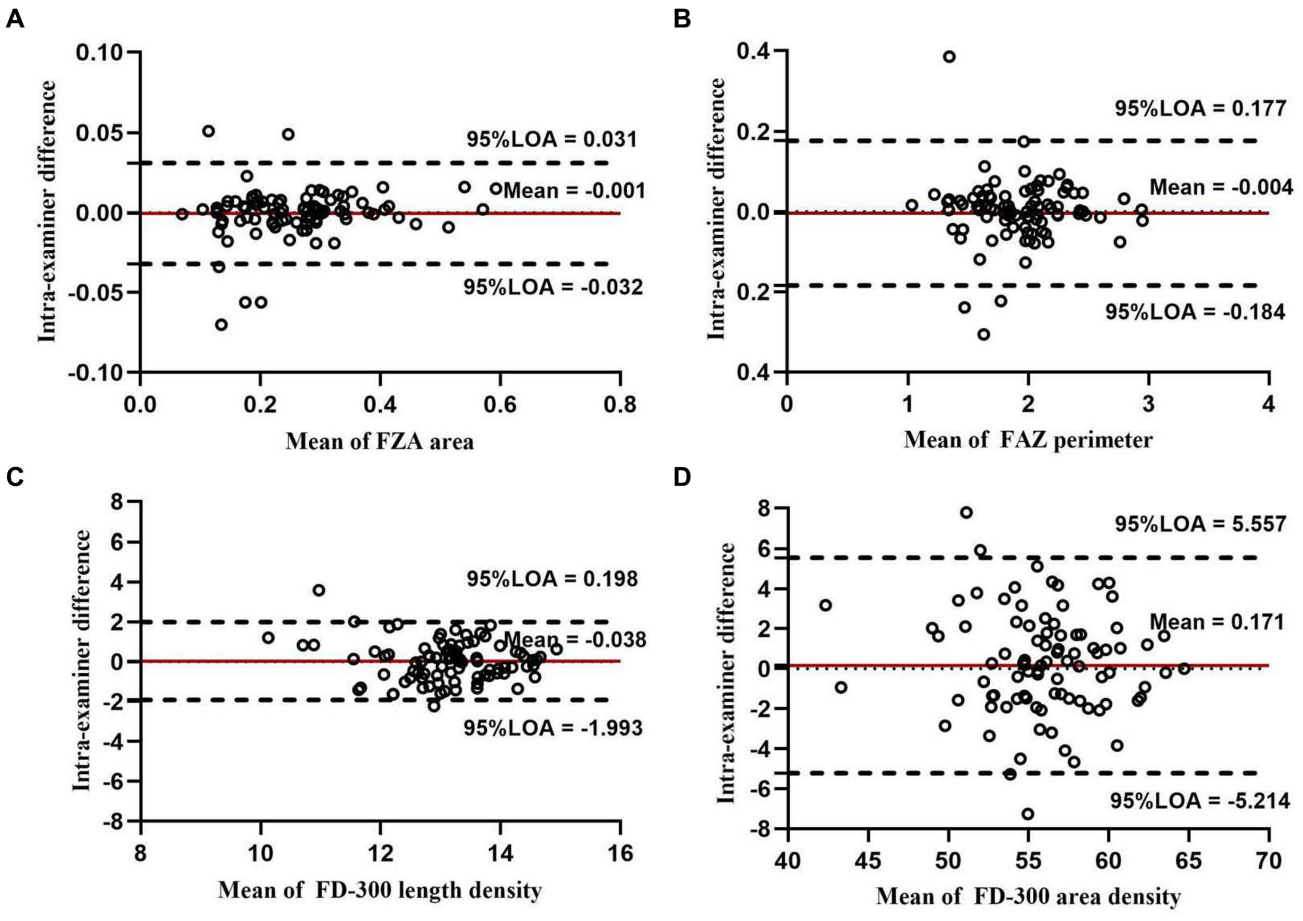
Bland–Altman plots showing the intra-examiner agreement for the parameters of FAZ and FD-300 in macula: agreement in FAZ area **(A)**, FAZ perimeter **(B)**, FD-300 length density **(C)**, and FD-300 area density **(D)**.

For FAZ-related parameters, the intra-examiner repeatability of FAZ and FD-300 is presented in Table 5. The FAZ and perimeter showed high repeatability (all COV values <0.01% and ICC = 0.981–0.991). The Bland–Altman plots (Figures 11A,B) also showed a good agreement. The FAZ acircularity index showed moderate repeatability.

**Table 5:** Intra-examiner variability of the parameters of FAZ and FD-300 in macular.

Section	Mean ± SD	COV (%)	ICC (95% CI)
FAZ area (mm^2^)	0.261 ± 0.103	<0.001	0.994 (0.991, 0.996)
FAZ perimeter (mm)	1.931 ± 0.382	0.207	0.987 (0.981, 0.992)
FAZ acircularity index	1.087 ± 0.032	<0.001	0.743 (0.611, 0.831)
FD-300 area density (%)	55.827 ± 4.060	7.229	0.862 (0.791, 0.909)
FD-300 length density (%)	13.075 ± 1.074	4.734	0.638 (0.449, 0.762)

FAZ, foveal avascular zone; FD-300, foveal VD 300.

For FD-300-related parameters, FD-300 area density and length density showed moderate reproducibility (COV = 4.734–7.229% and ICC = 0.638–0.862). The Bland–Altman plots (Figures 11C,D) also showed moderate agreement.

## Discussion

Many recent studies focused on the close relationship between children’s myopia and retinal vascular parameters via OCTA examination (12, 13). As an important research tool or a diagnostic tool, the repeatability and productivity of OCTA were significant and essential in children enrolled in this study. Ninety school children were enrolled in this study to evaluate the repeatability and reproducibility of OCTA measurements. The following two main findings of this study deserved our attention.

First, FAZ-related parameters exhibited excellent repeatability and reproducibility. Meanwhile, FD-300-related parameters showed moderate repeatability and reproducibility. In this study, the FAZ was 0.261 ± 0.104 mm^2^, a value within the range of the mean FAZ in healthy children (19–22) (from 0.23 to 0.30 mm^2^). The repeatability and reproducibility analysis showed that FAZ exhibited high reliability and precision in young children (COV < 0.001% and ICC = 0.994), which was higher than the repeatability values (COV = 7.6% and ICC = 0.97 in FAZ) found by Zhang et al. (16) in older children aged 11.51 ± 1.91 years. The repeatability in three FAZ parameters (all with COV values <0.210% and ICC = 0.743–0.994) in this study was also higher than the results (COV = 8.83–19.57% and ICC = 0.700–0.858) of the study by Pilar et al. (22). These two studies involved 6-to 15-year-old children but the refraction state of the children were different. Pilar et al. enrolled children with myopia, but this study included patients with emmetropia, myopia, and hyperopia. Previous studies compared peripapillary vascular parameters with macular parameters. The FAZ showed higher rates of repeatability. This study compared different macular VD layers. The SCP showed better reproducibility and repeatability. The FAZ showed the highest rates of repeatability and reproduction among all macular parameters, which was consistent with the findings of the study by Pilar et al. (10). The high reliability of the FAZ measurement in healthy children was similar to that found in healthy adults or in adults with ocular diseases (CoV < 6.5% and ICC = 0.957–0.997), (6, 8–10). These findings inferred that FAZ parameters exhibited good-to-excellent reliability in any population using OCTA, providing a precise evaluation and follow-up of any microvascular changes in the macular FAZ parameters for exploring the mechanism of myopia in children.

Second, macular VD in CC also exhibited excellent repeatability and reproducibility of OCTA measurement in children. VD measurement in SCP, ICP, and DCP showed moderate repeatability and reproducibility. These data (Tables 2, 4) revealed that, among four various plexus layers, the repeatability and reproducibility of VD measurement in the DCP were the worst, followed by ICP, and then by SCP, and CC, which exhibited the highest repeatability and reproducibility. The findings of this study showed that the repeatability of VD measurements varied depending on the depth and thickness of the retinal vascular plexus layers. A tendency found previously in other normal adult eyes (10) was in accordance with the current finding. The relatively worse repeatability for VD measurements for DCP and ICP inferred that the data about SCP and ICP VD relatively fluctuated, and their stability needs to be improved. Zhang et al. (19) reported the VD of CC in older children as 66.33% in the fovea and 66.29% in the parafovea, which was consistent with the results of this study. A good intra-examiner repeatability in VD measurement for CC (COV = 5.8% and ICC = 0.88) was reported in the study by Zhang et al. (19), which was less repeatable than that in the present study (COV = 1.00–6.10% and ICC = 0.856–0.950). Meanwhile, Zhang et al. (19) found that SCP and DCP had good intra-examiner repeatability (SCP: ICOV = 6.3%, CC = 0.91; DCP: COV = 7.1%, ICC = 0.82), a result better than that in this study. We believed that such a variation in results was because of the variation in the age of children and the various definitions of DCP. This study focused on the repeatability and reproducibility of different layers, areas, FAZ parameters, and FD-300 parameters in younger children.

Additionally, we also found that the repeatability of all evaluation indicators of OCTA was similar to the corresponding reproducibility. We found the lowest ICC value in the parafovea compared with the macula, fovea, and perifovea in the SCP, ICP, and DCP layers.

This study had some limitations. The size of the scan could be an interference factor for repeatability. This study exclusively utilized a 6 × 6 mm^2^ scan, which captured a larger area of information compared with the 3 × 3 mm^2^ scan. The repeatability of VD measurements in different retinal vascular plexus layers using the 3 × 3 mm^2^ scan in children needs further verification. This study included all refractive states, and the majority of patients had myopia. Further investigations are required to compare results among emmetropic, myopic, and hyperopic individuals. This study considered only Chinese children for VD measurements using OCTA. For more authentic results, VD measurements should be conducted using different techniques involving different ethnic groups.

In conclusion, OCTA was suggested as a noninvasive and reliable approach in clinical and scientific studies to investigate macular perfusion in children. The FAZ parameters and macular VD in CC exhibited the high repeatability and reproducibility of OCTA measurements in healthy children. The FAZ parameters and macular VD in CC could be widely and reliably applied in clinic. Also, further studies are needed to verify the results in young children. In different refractive states, VD measurements in ICP and DCP exhibited moderate repeatability and reproducibility, especially in the parafoveal region. Further studies should focus on improving VD measurements in ICP and DCP for more reliable and authentic results.

## Data availability statement

The original contributions presented in the study are included in the article/Supplementary material, further inquiries can be directed to the corresponding authors.

## Ethics statement

The studies involving human participants were reviewed and approved by Eye Hospital of Wenzhou Medical University. Written informed consent to participate in this study was provided by the participants’ legal guardian/next of kin. Written informed consent was obtained from the individual(s), and minor(s)’ legal guardian/next of kin, for the publication of any potentially identifiable images or data included in this article.

## Author contributions

KD, XH, MY, QW, JH, and RC contributed to conception and design of the study, JL, FF, HP, JY, YY, and WL organized the database. JH performed the statistical analysis. KD wrote the first draft of the manuscript. QW, JH, and RC wrote sections of the manuscript. All authors contributed to the article and approved the submitted version.

## Funding

This work was supported in part by the Science and Technology Planning Project of Zhejiang Province [LGF19H120002]; Project of National Natural Science Foundation of China (Grant No. 82271048); Shanghai Science and Technology (Grant No. 22S11900200); EYE & ENT Hospital of Fudan University High-level Talents Program (Grant No. 2021318); Multidisciplinary innovation team of traditional Chinese medicine for the treatment of myopia and amblyopia in children in Zhejiang Province. Program for Professor of Special Appointment (Eastern Scholar) at Shanghai Institutions of Higher Learning. The funders had no role in study design, data collection and analysis, decision to publish, or reparation of the manuscript.

## Conflict of interest

The authors declare that the research was conducted in the absence of any commercial or financial relationships that could be construed as a potential conflict of interest.

## Publisher’s note

All claims expressed in this article are solely those of the authors and do not necessarily represent those of their affiliated organizations, or those of the publisher, the editors and the reviewers. Any product that may be evaluated in this article, or claim that may be made by its manufacturer, is not guaranteed or endorsed by the publisher.
