# Supplementary material for: Inter-examiner and intra-examiner reliability of optical coherence tomography angiography in vascular density measurement of retinal and choriocapillaris plexuses in healthy children aged 6–15 years
Source: Front Med (Lausanne). 2023 Jun 1;10:1161942. doi: 10.3389/fmed.2023.1161942 (PMC10267442; doi:10.3389/fmed.2023.1161942)
Supplement: Supplementary file 1 [file Table_1.DOCX]

***Supplementary Material***

**Supplement table 1.** Inter-examiner variability

|  | | Parafovea | | | | Perifovea | | | |
| --- | --- | --- | --- | --- | --- | --- | --- | --- | --- |
|  | | Temporal | Superior | Nasal | Inferior | Temporal | Superior | Nasal | Inferior |
| SCP | Mean ± SD (%) | 52.740 ± 3.362 | 53.550 ± 3.343 | 51.449 ± 3.481 | 52.396 ± 3.745 | 46.808 ± 2.851 | 50.519 ± 3.067 | 54.582 ± 2.496 | 50.412 ± 2.627 |
|  | COV (%) | 12.35 | 9.94 | 13.11 | 12.34 | 7.83 | 6.04 | 5.17 | 4.56 |
|  | ICC (95% CI) | 0.589 (0.379, 0.729) | 0.686 (0.525, 0.793) | 0.611 (0.411, 0.743) | 0.697 (0.542, 0.800) | 0.712 (0.563, 0.811) | 0.809 (0.710, 0.875) | 0.704 (0.552, 0.805) | 0.803 (0.701,0.870) |
| ICP | Mean ± SD (%) | 49.889 ± 3.365 | 47.529 ± 3.675 | 49.639 ± 3.246 | 46.869 ± 4.053 | 47.344 ± 3.222 | 44.103 ± 4.636 | 43.370 ± 4.777 | 42.930 ± 4.726 |
|  | COV (%) | 9.61 | 14.83 | 9.47 | 15.82 | 10.95 | 15.41 | 21.38 | 22.77 |
|  | ICC (95% CI) | 0.734 (0.596, 0.825) | 0.648 (0.466, 0.768) | 0.716 (0.568, 0.813) | 0.707 (0.555, 0.806) | 0.670 (0.499, 0.783) | 0.815 (0.718, 0.878) | 0.747 (0.615, 0.833) | 0.718 (0.573, 0.814) |
| DCP | Mean ± SD (%) | 54.454 ± 5.768 | 54.048 ± 5.519 | 55.051 ± 5.903 | 55.153 ± 6.855 | 55.768 ± 5.160 | 56.154 ± 5.824 | 55.964 ± 6.194 | 57.605 ± 5.893 |
|  | COV (%) | 33.07 | 34.35 | 39.33 | 44.32 | 22.79 | 28.51 | 28.80 | 25.23 |
|  | ICC (95% CI) | 0.633 (0.442, 0.758) | 0.561 (0.335, 0.711) | 0.555 (0.323, 0.708) | 0.653 (0.472, 0.772) | 0.690 (0.529, 0.796) | 0.694 (0.536, 0.799) | 0.738 (0.601, 0.828) | 0.737 (0.601, 0.827) |
| CC | Mean ± SD (%) | 68.853 ±4.074 | 67.685 ±4.087 | 67.282 ± 4.146 | 69.129 ±3.860 | 72.198 ± 2.748 | 71.158 ±2.807 | 69.883 ±3.035 | 71.154 ±3.129 |
|  | COV (%) | 4.57 | 5.30 | 6.06 | 4.67 | 1.74 | 2.20 | 2.10 | 2.14 |
|  | ICC (95% CI) | 0.896 (0.843, 0.9323) | 0.881 (0.820, 0.9223) | 0.867 (0.797, 0.9123) | 0.879 (0.817, 0.9203) | 0.911 (0.864, 0.9413) | 0.891 (0.835, 0.9293) | 0.914 (0.87,0 0.9443) | 0.917 (0.874, 0.9453) |

SCP: superficial capillary plexus; ICP: intermediate capillary plexus; DCP: deep capillary plexus; CC: choroidal capillary plexus.

**Supplement table 2.** Intra-examiner variability

|  | | Parafovea | | | | Perifovea | | | |
| --- | --- | --- | --- | --- | --- | --- | --- | --- | --- |
|  | | Temporal | Superior | Nasal | Inferior | Temporal | Superior | Nasal | Inferior |
| SCP | Mean ± SD (%) | 53.08 ± 2.95 | 53.94 ± 3.13 | 51.76 ± 3.00 | 52.74 ± 3.53 | 46.97 ± 2.57 | 50.69 ± 2.80 | 54.75 ± 2.26 | 50.57 ± 2.52 |
|  | COV (%) | 11.20 | 11.19 | 11.26 | 10.23 | 5.47 | 5.72 | 4.60 | 5.65 |
|  | ICC (95% CI) | 0.489 (0.222, 0.664) | 0.559 (0.330, 0.709) | 0.529 (0.283, 0.691) | 0.727 (0.585, 0.820) | 0.762 (0.638, 0.843) | 0.773 (0.656, 0.851) | 0.678 (0.510, 0.788) | 0.711 (0.561, 0.810) |
| ICP | Mean ± SD (%) | 49.712 ± 3.201 | 47.428 ± 3.550 | 49.550 ± 3.189 | 46.987 ± 3.898 | 47.344 ± 3.198 | 44.184 ± 4.913 | 43.442 ± 4.877 | 43.341 ± 4.955 |
|  | COV (%) | 11.12 | 11.01 | 8.50 | 15.41 | 9.08 | 12.48 | 13.90 | 19.70 |
|  | ICC (95% CI) | 0.624 (0.431, 0.752) | 0.733 (0.596, 0.824) | 0.738 (0.603, 0.827) | 0.689 (0.528, 0.795) | 0.736 (0.599, 0.826) | 0.873 (0.807, 0.916) | 0.849 (0.770, 0.900) | 0.792 (0.685, 0.863) |
| DCP | Mean ± SD (%) | 54.401 ± 5.752 | 54.213 ± 5.454 | 55.132 ± 5.969 | 55.073 ± 6.853 | 55.515 ± 5.333 | 56.100 ±6.012 | 55.968 ±6.439 | 57.128 ± 6.086 |
|  | COV (%) | 21.63 | 24.45 | 25.15 | 30.54 | 17.59 | 21.65 | 23.81 | 21.84 |
|  | ICC (95% CI) | 0.785 (0.673, 0.858) | 0.716 (0.568, 0.813) | 0.761 (0.637, 0.843) | 0.785 (0.673, 0.859) | 0.795 (0.689, 0.865) | 0.799 (0.695, 0.868) | 0.811 (0.713, 0.876) | 0.799 (0.695, 0.868) |
| CC | Mean ± SD (%) | 68.678 ± 4.063 | 67.467 ± 4.082 | 67.073 ± 4.024 | 69.046 ± 4.007 | 72.147 ± 2.691 | 71.097 ± 2.805 | 69.799 ± 2.986 | 71.109 ± 3.129 |
|  | COV (%) | 4.36 | 3.95 | 4.70 | 5.40 | 1.65 | 1.32 | 1.78 | 2.06 |
|  | ICC (95% CI) | 0.901 (0.850, 0.935) | 0.913 (0.868, 0.943) | 0.894 (0.838, 0.930) | 0.868 (0.799, 0.913) | 0.912 (0.866, 0.942) | 0.937 (0.905, 0.959) | 0.926 (0.888, 0.951) | 0.920 (0.879, 0.947) |

SCP: superficial capillary plexus; ICP: intermediate capillary plexus; DCP: deep capillary plexus; CC: choroidal capillary plexus.
